# Environmentally Compatible Lead-Free Perovskite Solar Cells and Their Potential as Light Harvesters in Energy Storage Systems

**DOI:** 10.3390/nano11082066

**Published:** 2021-08-15

**Authors:** Il Jeon, Kyusun Kim, Efat Jokar, Minjoon Park, Hyung-Woo Lee, Eric Wei-Guang Diau

**Affiliations:** 1 Department of Chemistry Education, Graduate School of Chemical Materials, Crystal Bank Institute, Pusan National University, Busan 46241, Korea; ewrb23@gmail.com; 2 Department of Applied Chemistry and Institute of Molecular Science, National Yang Ming Chiao Tung University, Hsinchu 30010, Taiwan; efatjokar@gmail.com; 3 Center for Emergent Functional Matter Science, National Yang Ming Chiao Tung University, Hsinchu 30010, Taiwan; 4 Research Center of Energy Convergence Technology, Department of Nanoenergy Engineering, Pusan National University, Busan 46241, Korea; mjpark@pusan.ac.kr (M.P.); LHW2010@pusan.ac.kr (H.-W.L.)

**Keywords:** lead-free perovskite solar cells, tin perovskite, thin-film solar cells, energy storage systems, photo-charging batteries

## Abstract

Next-generation renewable energy sources and perovskite solar cells have revolutionised photovoltaics research and the photovoltaic industry. However, the presence of toxic lead in perovskite solar cells hampers their commercialisation. Lead-free tin-based perovskite solar cells are a potential alternative solution to this problem; however, numerous technological issues must be addressed before the efficiency and stability of tin-based perovskite solar cells can match those of lead-based perovskite solar cells. This report summarizes the development of lead-free tin-based perovskite solar cells from their conception to the most recent improvements. Further, the methods by which the issue of the oxidation of tin perovskites has been resolved, thereby enhancing the device performance and stability, are discussed in chronological order. In addition, the potential of lead-free tin-based perovskite solar cells in energy storage systems, that is, when they are integrated with batteries, is examined. Finally, we propose a research direction for tin-based perovskite solar cells in the context of battery applications.

## 1. Introduction

**Figure 1:**
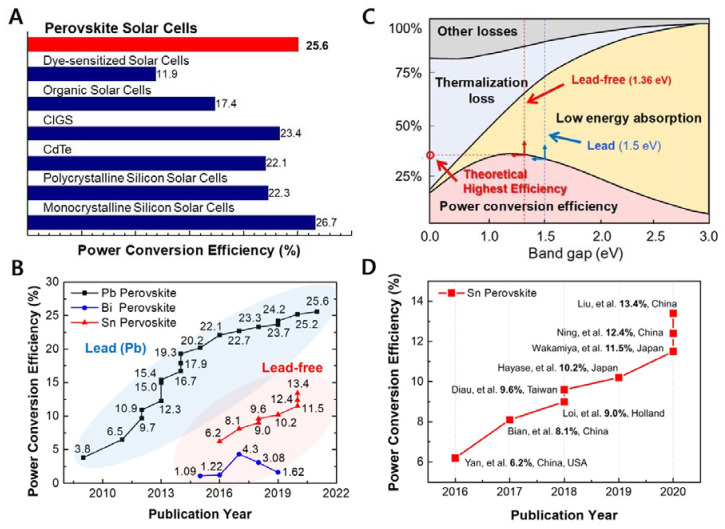
(**A**) Bar graph showing the highest certified PCEs of the different types of photovoltaics. (**B**) Reported PCEs of Pb-based, Bi-based, and Sn-based PSCs from the initial stage of development to date. (**C**) Shockley–Queisser limit graph showing the PSC type that has a relatively high ideal bandgap. (**D**) PCE chart showing the development of Sn-PSCs.

Since a pioneering report by Kojima et al., in 2009 [1] and a subsequent breakthrough by Kim et al. and Lee et al. [2,3] metal halide perovskite solar cells (PSCs) have substantially transformed the field of photovoltaics. Such next-generation light harvesters exhibit an exceptionally high-power conversion efficiency (PCE) owing to the long exciton diffusion length [4], high absorption coefficient [5], and defect tolerance [6] of perovskite materials. Presently, the highest PCE certified by the National Renewable Energy Laboratory (NREL) is 25.6% [7,8,9,10]. This value is higher than that of other types of photovoltaics and extremely close to that of monocrystalline silicon solar cells (Figure 1A). With rapid and substantial improvements in PSC technology, the intrinsic device instability of PSCs has also been greatly improved to the level of commercialization [11,12]. However, the presence of lead (Pb) in perovskite materials lowers the market value of PSCs, as Pb is perceived to be toxic and detrimental to the environment. The use of Pb in electronic products is banned by the Restriction of Hazardous Substances (RoHS) directive in Europe [13,14,15]. According to the World Health Organization (WHO), the human body cannot purge Pb through metabolism, and children are at a particularly high risk of Pb poisoning [16,17]. Moreover, Pb can easily spread via airborne particles into the air, water, and soil [18,19]. Thus, the scientific community has been searching for Pb alternatives. Metals such as tin (Sn), bismuth (Bi), antimony (Sb) have been reported to form metal halide perovskite crystals, and their application in PSCs has been proven to be effective (Figure 1B) [20,21]. Sn-based PSCs (Sn-PSCs) have thus far shown the best prospects. Although there are fewer reports on Sn-PSCs compared with those on Pb-PSCs, the PCE and stability of Sn-based PSCs have been improved rapidly. In addition, the ideal bandgap of Sn-based perovskite materials plays a crucial role (Figure 1C). Sn-based perovskites such as methylammonium tin iodide (MASnI_3_), formamidinium tin iodide (FASnI_3_), and caesium tin iodide (CsSnI_3_) have direct bandgaps of approximately 1.20, 1.41, and 1.3 eV, respectively, which are narrower than those of Pb-based perovskites [22]. However, Sn^2+^ in Sn-based perovskite is prone to form Sn^4+^. Although the product of this oxidation reaction is SnO_2_, which is environment friendly, it dramatically lowers the device stability. This review focuses on Sn-PSCs that have significantly contributed to the development of Sn-PSC technology thus far (Figure 1D). Chronological accounts of Sn-PSCs from their initial development to the latest certified PCE can shed light on future prospects and research directions. Furthermore, this work reviews papers on solar cell–battery integration technologies to assess the potential of lead-free Sn-based PSCs as light harvesters in energy storage systems (ESSs).

## 2. Development of Pb-Free Sn-Based Perovskite Solar Cells

### 2.1. Pb-Free Sn-Based Perovskite Solar Cells at Their Infancy

**Figure 2:**
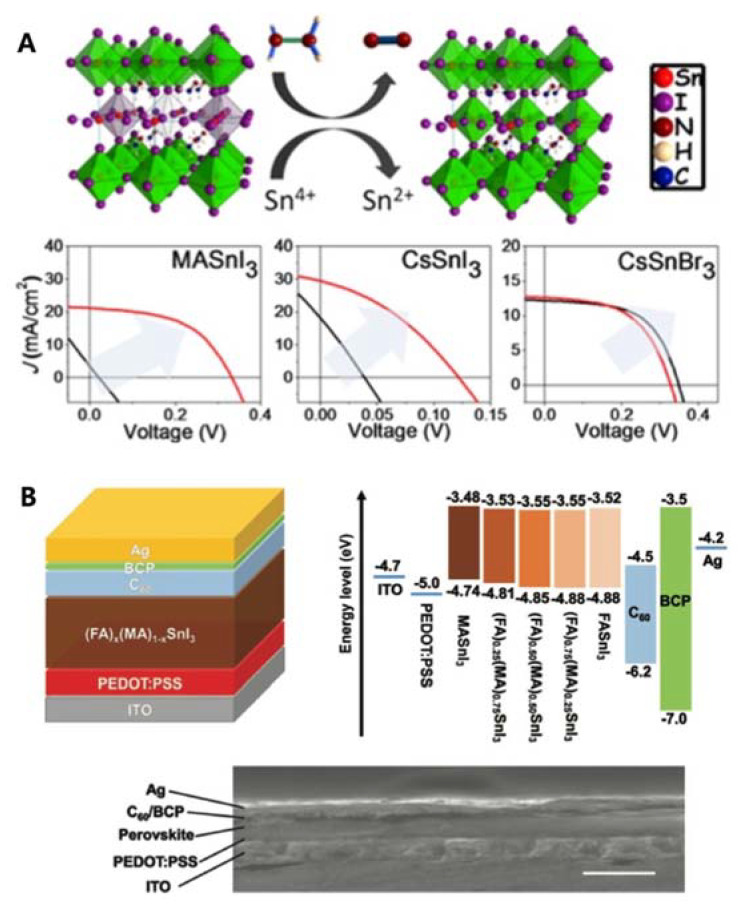
(**A**) Proposed mechanism of hydrazine vapour reaction with Sn-based perovskite materials (reduction process: 2SnI_6_^2−^ + N_2_H_4_ → 2SnI_4_^2−^ + N_2_ + 4HI). *J*–*V* curves for MASnI_3_ solar cells, CsSnI_3_ solar cells, and CsSnBr_3_ solar cells with and without hydrazine vapour concentrations. Reprinted with permission from ref. [27] Copyright 2017, The American Chemical Society. (**B**) Schematic of the device structure, band alignment diagram, and a cross-sectional scanning electron microscope image of a completed device (scale bar: 500 nm). Reprinted with permission from ref. [30]. Copyright 2017, the John Wiley and Sons.

The first Sn-PSCs were reported by Kanatzidis and co-workers in 2014 [23]. The same year, Snaith and co-workers reported a higher PCE of 6.40% using the same device configuration [24]. However, they used MASnI_3_ as the active layer in an n-i-p structure, of which the device performance was difficult to reproduce due to the poor stability nature of this device configuration [25]. The first inverted-type (p-i-n) Sn-PSCs were demonstrated by Yan and co-workers. They used poly(3,4-ethylenedioxythiophene) polystyrene sulfonate (PEDOT:PSS) as the hole-transporting layer, and C_60_ and bathocuproine (BCP) as the electron-transporting layers [26]. Thus, a PCE of 6.22% was obtained (Table 1A). The key improvement was the addition of SnF_2_ during diethyl ether antisolvent dripping, which resulted in highly uniform and pinhole-free compact FASnI_3_ perovskite films. Kanatzidis and colleagues deepened the understanding of Sn-PSCs by including Cs in Sn-based perovskite fabrication [27]. Through the reduction of a vapour atmosphere during the preparation of Sn-based perovskites, PCEs of 3.89%, 1.83%, and 3.04% for MASnI_3_, CsSnI_3_, and CsSnBr_3_, respectively, were obtained. Furthermore, the device stability and performance were considerably improved when the Sn^4+^/Sn^2+^ ratio was reduced by 20% (Table 1B, Figure 2A). Seok and co-workers reported a PCE of 4.8%, which remained stable for more than 100 d (Table 1C) [28]. The key to obtaining such a high device stability was the use of a SnF_2_-pyrazine complex, which could disperse into the perovskite film better than SnF_2_ alone. Kanatzidis and co-workers also reported a new type of hollow Sn-based perovskite [29], in which ethylenediammonium (en) served as the A-site cation in a FASnI_3_ perovskite structure, forming {en}FASnI_3_, a new hybrid perovskite structure. They showed that changing the A-site cation significantly increased the bandgap compared to X-site anion tuning, owing to the unique ability of en to create Schottky defects in perovskites. A relatively high PCE of 7.14% was attained with a device stability time of over 1000 h (Table 1D). The Sn-PSCs in the initial stage of development report PCEs under 8% despite the gradual improment over time. They rely on single A-site cation system until the work by Ke et al. [29], which demonstate en-added Sn perovskite. However, the amount of en was too small to regard the the perovskite composition as a mixed cation. Rather, en served more as an additive than a cation.

### 2.2. Burgeoning of Sn-Based Perovskite Solar Cells via Mixing Cations in Inverted Architecture

Later, an even higher PCE of 8.12% was reported by Huang and colleagues using a mixed organic cation as the perovskite photoactive material, (FA)_x_(MA)_1−x_SnI_3_ in an inverted structure (Table 2E, Figure 2B) [30] for the first time. Through optimisation of the FA and MA ratios, FA_0.75_MA_0.25_SnI_3_ exhibited a relatively high PCE due to a high open-circuit voltage (*V*_OC_) of 0.61 V arising from an improved perovskite film morphology and energy level alignment. Further, in their devices, 10 mol% of SnF_2_ was added to the optimised FA and MA mixed cations, which helped their PCE to reach above 8%. The obtained *V*_OC_ was the highest among the values reported for Sn-PSCs.

#### 2.2.1. A-Site Cation Engineering in Sn-Based Perovskite Solar Cells

Sn-based perovskites have excellent prospects to replace Pb, yet the easy oxidation and the low formation energy of Sn vacancies of Sn^2+^ serve as a great hindrance to high PCE. One of the strategies to overcome this is engineering the A-site cation of ASnI_3_. Mixing the A-site cations not only improve the film quality, such as morphology, but it can also change the optoelectrical properties of the Sn perovskites. This is because the perovskite crystal structure of ABX_3_ is determined chiefly by the size of the A-site cation. Therefore, A-site cation engineering was used initially to fine tune the bandgap and to change the chemical composition of the Sn perovskite precursors. Initially, researchers mixed MA^+^, FA^+^, and Cs^+^ A-site cations. The candidates extended to HA, EA, GA, EDA, TN, BEA, PN, BA, HEA, 4AMP, PEA, PPA, and 5-AVA in the later stage of the Sn-PSC evolution.

Loi and co-workers reported a method for lowering the background carrier density arising from the intrinsic defects in Sn perovskites. Sn vacancies and Sn^4+^ were reduced by more than one order of magnitude via the deposition of near-single-crystalline FASnI_3_, which possesses the orthorhombic a-axis in the out-of-plane direction [31]. This was achieved by mixing a small amount of a layered two-dimensional Sn perovskite (0.08 M) with 0.92 M of three-dimensional FASnI_3_. The fabricated devices had a PCE of 9.0%, with negligible hysteresis and light soaking owing to a low trap density and efficient charge collection. SnF_2_ was used as a reducing agent, as recommended in previous reports (Table 3F).

### 2.3. Sequential Deposition Technique as the Game Changer

The Jen group reported a sequential deposition method using trimethylamine (TMA) as a Lewis base to form SnI_2_- and SnF_2_-TMA complexes in the first deposition step, and these were intercalated with FAI to obtain FASnI in the following step [32]. TMA facilitated the formation of homogeneous SnI_2_ and SnF_2_ layers. Later, the Diau group realised a hybrid solvent system used in the second step of sequential deposition to control the film morphology [33]. The principle entails the formation of an iodoplumbate anion intermediate phase, which mediates perovskite crystallisation as a Lewis acid–base adduct, between metal halides (Lewis acid) and polar aprotic solvents (Lewis base). The Lewis acid–base adduct formed during the SnI_2_ deposition step controlled the volume expansion and promoted rapid reaction with MAI and FAI. Thus, denser and more compact FASnI_3_ films with larger crystalline domains (>1 μm) than those of the previously reported Sn-based perovskite films were formed. As a result, a high PCE of 7.09% with a good device stability was obtained (Table 4G).

### 2.4. Doping Sn Perovskites Using Additives

**Figure 3:**
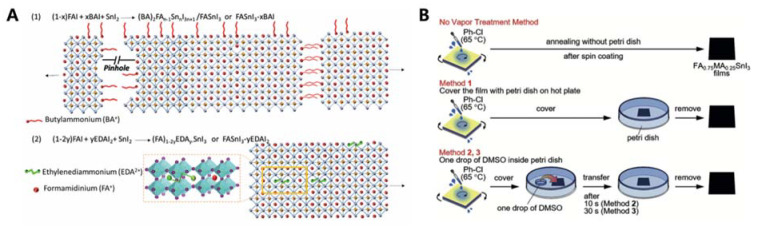
(**A**) Schematic representations of perovskite crystals in the presence of BAI and EDAI_2_ additives. Reprinted with permission from ref. [34] Copyright 2018, the Royal Society of Chemistry. (**B**) Illustration of the vapour modification method discussed in this review. Reprinted with permission from ref. [36]. Copyright 2018, the John Wiley and Sons.

**Figure 4:**
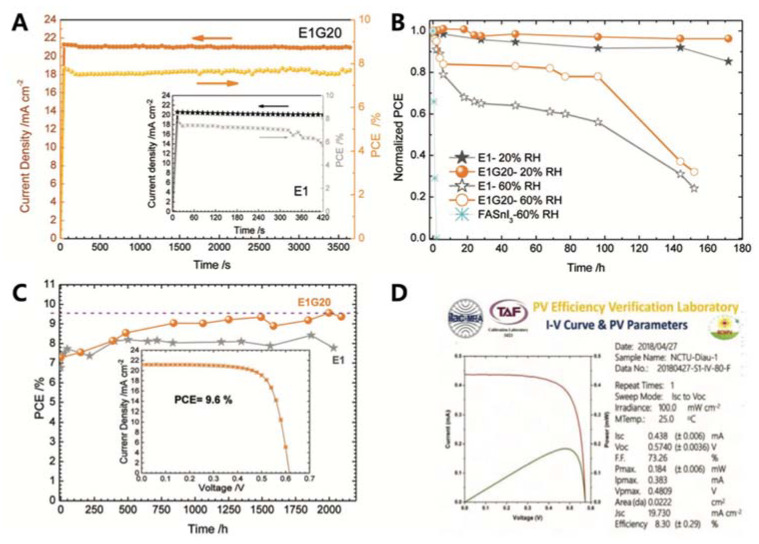
(**A**) Stabilised efficiency of power conversion and photocurrent density of two different devices tested in different environments. The measurement was taken at the position of maximum power for 1 sun irradiation with an AM1.5G solar simulator for 3600 s. Device reliability test conducted in (**B**) ambient air with relative humidity = 20% and 60% without encapsulation and (**C**) in N_2_-filled glove box. (**D**) Efficiency of 8.30%, certified by ISO-approved PV Efficiency Verification Laboratory in Taiwan. Reprinted with permission from ref. [38] Copyright 2018, the John Wiley and Sons.

Diau and co-workers studied the doping effect of ethylenediammonium diiodide (EDAI_2_) and butylammonium iodide (BAI) on organic cations, which passivated the defect sites and improved the film morphology and perovskite crystallinity [34]. The addition of BAI changed the orientation of the perovskite crystal growth and enhanced the connectivity of the crystal grains. The existing pinholes were passivated by the addition of EDAI_2_. This prevented the oxidation of Sn^2+^ to Sn^4+^ and promoted slow relaxation of the crystal structure, as evidenced by X-ray diffraction, X-ray photoelectron spectroscopy, and photoluminescence decay measurements. With the addition of EDAI_2_ (1%), the initial PCE of 7.4%, obtained for the FASnI_3_-based PSC, increased to 8.9% after ageing. The resulting devices retained their PCE for more than 2000 h (Table 5H and Figure 3A). Hayase and co-workers achieved highly stable and efficient Sn-PSCs by adding Ge to a Sn-based perovskite precursor. The Ge–Sn mixed perovskite film exhibited a bandgap between 1.4 to 1.5 eV, based on photoacoustic spectroscopy results [35]. The amount of added Ge was optimised at 5%, and thus, the perovskite structure was FA_0.75_MA_0.25_Sn_1−x_GexI_3_, as confirmed by X-ray diffraction and X-ray photoelectron spectroscopy measurements. The initial PCE of 4.48% increased to 6.90% after 72 h of ageing. The devices underwent measurements outside a glove box, without encapsulation (Table 5H). Subsequently, two simple methods for improving Sn-PSCs were proposed in a report by Liu et al. [36], The first method involved the use of a high-temperature antisolvent for full coverage of the Sn-based perovskites on PEDOT:PSS. The second method entailed annealing under a low partial pressure of dimethyl sulfoxide vapour, which increased the perovskite crystallite domains. A PCE of 7.20% was obtained owing to topographical and electrical improvements (Table 5J and Figure 3B). Diau and co-workers achieved a PCE close to 10% in an inverted-type Sn-PSC, which used a mixture of nonpolar organic cation, guanidinium (GA^+^), and formamidinium (FA^+^) [37]. FASnI_3_ was fabricated in the presence of 1% EDAI_2_ as an additive, with an optimised ratio of GAI to FAI of 20:80. This composition resulted in a PCE of 8.5%, which then increased to 9.6% after storage inside a glove box (Table 5K). The devices were stable under continuous 1 sun illumination for 1 h without encapsulation. A certified PCE of 8.3% was obtained, which was the highest to the best of our knowledge (Figure 4). Han and colleagues reduced the crystallisation speed of Sn-based perovskites by adding poly (vinyl alcohol) (PVA) which induced hydrogen bonding interactions during the growth of FASnI_3_ [38]. This reduced the number of defect sites, which was reflected by the increased *V*_OC_. The recorded *V*_OC_ of 0.63 V is ascribed to the suppressed migration of iodide ions in the presence of the PVA additive. A PCE of 8.92% was attained, and the device exhibited stable operation for 400 h (Table 5L).

### 2.5. High Performance All-Inorganic Sn Perovskite Solar Cells

Padture and co-workers [39] demonstrated all-inorganic Sn-PSCs that showed high unprecedentedly PCE. The addition of Ge to CsSnI_3_ formed CsSn_0.5_Ge_0.5_I_3_, which is highly stable in air, owing to the favourable Goldschmidt tolerance (0.94) and octahedral factor (0.4) of the inorganic Sn-based perovskite films. The high oxidation activity exhibited by Ge was due to the formation of an ultrathin (<5 nm) native oxide protective film on the surface of the CsSn_0.5_Ge_0.5_I_3_ perovskite. Owing to the inorganic nature of the film, the stability was greater than that of MAPbI_3_. A PCE of 7.11% was obtained from the Sn-PSCs, and the device was stable for more than 500 h of continuous operation (Table 6M).

### 2.6. Anti-Oxidation as the Key to Improving V_OC_ and Device Stability

**Figure 5:**
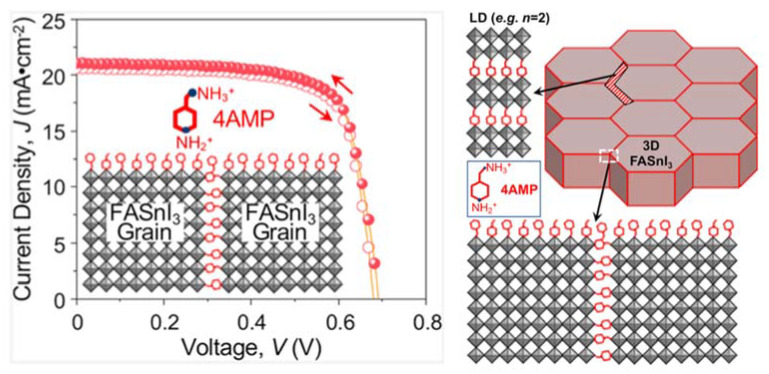
Schematic illustration of the optimum 4AMP functionalisation of grain surfaces and grain boundaries in FASnI_3_ perovskite polycrystalline thin film, together with the *J–V* curves of the devices showing negligible hysteresis. Reprinted with permission from ref. [42] Copyright 2020, the American Chemical Society.

Hayase and co-workers reported a PSC with a PCE surpassing 10% [40]. They suspected the low PCE of Sn-based PSCs to their *V*_OC_ values, which were lower than those of Pb-based PSCs. The cause for the low *V*_OC_ values was the presence of defects and trap sites, which were exacerbated by the oxidation of Sn^2+^ to Sn^4+^ in air. To address this issue, the Sn-based perovskites were treated with an ethylenediamine (EDA) Lewis base to form FA_0.98_EDA_0.01_SnI_3_. X-ray photoelectron spectroscopy revealed that the recombination reaction originated from the nonstoichiometric Sn:I ratio and not the Sn^4+^:Sn^2+^ ratio. A PCE of 10.18% was recorded (Table 7N). In 2020, Han and colleagues reported an inverted-type PSC with a PCE of 9.47% (Table 7O) [41], wherein an anti-oxidising capping layer was formed on the surface of FASnI_3_ to prevent oxygen attacks. Further, 4-fluorobenzohydrazide (FBH), along with chlorobenzene, was added during the anti-solvent dripping step. Thus, this perovskite composition was written as FASnI_3_-FBH. This enabled the fabrication process to be performed even when the oxygen content was 100 ppm. Thus, the device stability was considerably enhanced. In addition, through solvent engineering and optimisation, large crystal grains were obtained with fewer Sn^4+^ defects and long carrier recombination lifetime. Thus, high-performance Sn-PSCs with a long device lifetime of 600 h under illumination by light soaking (AM 1.5G, 100 mW cm^2^) were achieved. Paditure and co-workers applied a new hole-transporting layer, Cu-doped NiO_x_ to fabricated inverted-type PSCs [42]. Further, the Sn-based perovskite layer was also modified. They introduced 4-(aminomethyl)-piperidinium (4AMP) into FASnI_3_ to form FASnI_3_-4AMP. This incorporation enhanced the electrical properties and material durability. The bulky divalent organic cation, 4AMP, surrounded the grain boundaries of the three-dimensional Sn-based perovskite crystals, protecting the grain from oxygen intrusion while improving connectivity. The 4AMP in between the domains was visible in the scanning electron microscopic images. The fabricated devices exhibited the highest PCE of 10.9% with negligible hysteresis (Table 7P and Figure 5). The device exhibited stable operation for approximately 500 h. In the same year, Han and co-workers reported inverted-type Sn-PSCs with amorphous-polycrystalline CsFASnI_3_ [43]. Owing to the added Cs, Sn-based perovskites with this particular composition to block oxygen and moisture attacks as well as to suppress ion diffusion within the fabricated devices. Moreover, the energy levels were better aligned compared to the charge extraction, and transport became more efficient. The device exhibited a high PCE of 10.18% with minor hysteresis. Its efficiency was 10.08%, as verified by Newport Laboratory, USA, with greater-than-95% PCE retention after 1000 h of operation (Table 7Q). Wakamiya and co-workers reported that Sn(0) could scavenge Sn(IV) impurities in a precursor solution [44]. This treatment prolonged the lifetime of the FA_0.75_MA_0.25_SnI_3_-based PSCs and induced strong PL and charge collection. Further, the selective reaction between the dihydropyrazine derivative and added SnF_2_ was promoted. The mechanism of the reactions with Sn has been corroborated by various analyses. Under this new nanoparticle regime, an impressive PCE of 11.5% together with *V*_OC_ of 0.76 V were obtained (Table 7R). The devices exhibited a record-high *V*_OC_ of 0.76 V among those of the previously reported Sn-PSCs. Thus, this seminal work has set records in many aspects. Energy-level alignment plays a vital role in increasing *V*_OC_.

### 2.7. Energy Level Alignment as the Key to Improving V_OC_

**Figure 6:**
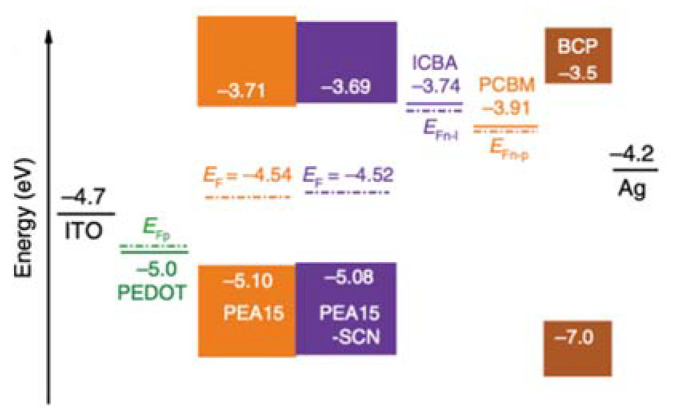
Schematic illustration of energy levels with dashed lines representing the quasi-Fermi level of ICBA (E_Fn–I_), phenyl-C61-butyric acid methyl ester (PCBM) (E_Fn–P_), and PEDOT:PSS (E_Fp_). Reprinted with permission from ref. [50]. Copyright 2020, the Nature Publishing Group.

Energy level alignment plays a vital role in increasing *V*_OC_. Diverse fullerene derivatives have been used to improve *V*_OC_ [45,46,47,48,49]. Among them, Ning and colleagues used indene-C_60_ bisadduct (ICBA) fullerene as an electron-transporting layer in inverted-type Sn-PSCs [50]. ICBA is a commercially available mainstream fullerene derivative; thus, it is laudable that they acquired such a high result using an easily accessible material. Furthermore, the obtained PCE of 12.4% has reached a new milestone surpassing the 12% limit (Table 8S). In addition to the electron transporting layer, a new Sn-based perovskite film was adopted. The authors incorporated C_6_H_5_CH_2_CH_2_NH_3_I (PEAI) and NH_4_SCN (SCN) into the FASnI_3_-based perovskite film to match with the charge transport layers (Figure 6). The energetically optimised device produced a *V*_OC_ of 0.94 V, which is the highest *V*_OC_ reported among Sn-PSCs to date. Hayase and colleagues [51] improved their previous device and reported a remarkable device performance of 13.24% (Table 8T). The energy level of the Ge-doped FA_0.98_EDA_0.01_SnI_3_ film was controlled by tuning the amount of ethylammonium (EA), which led to a considerable enhancement in *V*_OC_. The obtained *V*_OC_ of 0.84 V was not as high as 0.94 V, reported by Ning and colleagues [50], however, the overall PCE was higher for the former than for the later due to a relatively higher short-circuit current density (*J*_SC_) and fill factor (FF) caused by the higher perovskite film quality, as proven by various measurements, such as space charge limited current (SCLC) measurements. In addition to the one-step method applied for most of the studies reported herein, Diau and co-workers [52] developed a two-step method of solution processing to fabricate 3D/2D hybrid Sn-based perovskite films. Based on the perovskite structure, FA_0.8_GA_0.2_SnI_3_, where GA stands for guanidinium, various bulky ammonium cations (BACs) and hexafluoro-2-propanol were applied to form a 2D Sn-based perovskite layer. Among the various BACs, anilinium was found to form the thinnest 2D layer on the surface, which led to the best PCE of 10.6% with a self-healing effect (Table 8U). Recently, Han and colleagues developed a low-temperature deposition method with an additive of PEAI or post-treatment by n-propylammonium iodide (PAI) to grow large crystal grains. [53]. Thus, the quality of Sn-based perovskites was improved by slowing down the crystal growth or inducing a preferential orientation. For example, Lewis bases (e.g., dimethyl sulfoxide (DMSO), trimethylamine, and thiocyanate) formed Lewis adducts with SnI octahedra to slow down the crystallisation. In addition, hydrogen bonding was found to have the same effect as that of crystal orientation. Furthermore, long-chain organic ammonium (e.g., phenylethylammonium (PEA), butylammonium (BA), and EDA (or en) was used to assist the oriented growth of Sn-based perovskites by restricting the tilting of the grains). They harnessed a high-temperature anti-solvent to obtain a highly dense perovskite film. Moreover, the precursor solution was cold, and this decreased the number of nuclei and decelerated the nucleation rate by increasing the Gibbs free energy. This also entailed an ordered arrangement of perovskite nuclei along the energetically favourable <h00> crystalline directions. Furthermore, the crystal growth was retarded by torpid solute diffusion and solvent evaporation, promoting Ostwald ripening. In addition, the authors used n-propylammonium iodide (PAI) for post-treatment to maximise the device performance. A PCE of 12.11% was obtained, and the device was stable for 500 h (Table 8V).

### 2.8. Lead-Free Sn PSCs as Photo-Charging Power Source 

**Figure 7:**
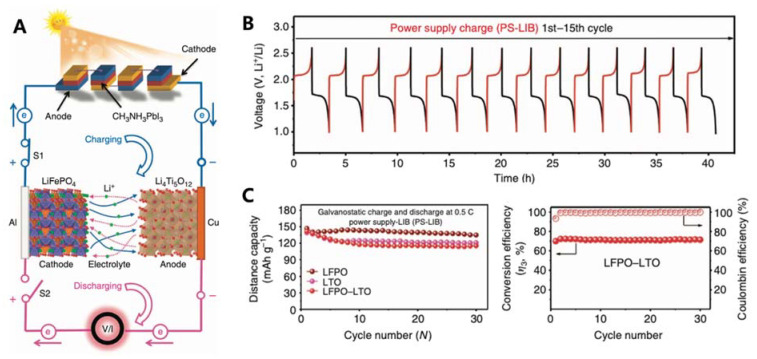
(**A**) Schematic of a fabricated system of LIB photo-charging by PSCs. (**B**) V–t curves of a fresh LIB cell measured at 0.5 C in the voltage range of 1.0–2.6 V for 15 cycles. (**C**) Cycling performance of different types of LIB cells measured at 0.5 C in the voltage ranges of 1.0–3.0, 2.5–4.0 and 1.0–2.6 V for 30 cycles. Reprinted with permission from ref. [54] Copyright 2015, the Nature Publishing Group.

Electric vehicles that use lithium-ion batteries (LIBs) have attracted significant interest. LIBs have high energy density and does not have the memory effect. Suffering from small self-discharge is another advantage of LIBs. The performance of LIBs is determined by stable charging and discharging of the current density over copious cycles. Parameters like the battery state of charge (BSOC), defined by the fraction of the total energy or battery capacity discharged over the total available is used a one of the parameters. One way to augment the performance of batteries in electric cars is via the application of self-charging suppliers. This can be achieved in numerous ways, one of which is the installation of a solar roof. Thin-film perovskite photovoltaics are attractive as they can directly photo-charge LIBs. However, no study has been conducted on the integration of Sn-PSCs and batteries, and only a few reports on the photo-charging of Pb-based PSCs are available. In this section, we examine these publications and elucidate the potential of Sn-PSCs using a similar approach. In 2015, Xu et al. [54], connected four single MAPbI_3_-based PSCs in series with an LIB assembled with a LiFePO_4_ cathode and a Li_4_Ti_5_O_12_ anode. The integration yielded a photoelectric conversion and storage efficiency of 7.80% with adequate cycling stability (Figure 7). A PCE of 12.65% was achieved when all four cells were connected. Each cell exhibited an average PCE of 15.67%. At 0.5 C, the PSC–LIB exhibited a capacity retention rate similar to that of galvanostatically charged and discharged LIBs with a conventional power supply, when measured from 0.1 C to 1 C. In 2017, a simple and efficient photo-charging design, which used a DC–DC voltage boost converter between a PSC and a Li_4_Ti_5_O_12_-LiCoO_2_ Li-ion cell, was proposed by Gurung et al. [55]. The converter boosted the low input voltage of the PSC to charge the lithium-ion cells more effectively and offer overvoltage protection. This approach resulted in an efficiency of 9.36% and an average storage efficiency of 77.2% at 0.5 C discharge. The high charging efficiency of the PSC–LIB was attributed to the high PCE of the PSCs and low potential polarisation between the charge and discharge voltage plateaus of the Li_4_Ti_5_O_12_–LiCoO_2_ Li-ion cell. Li et al. [56] demonstrated a system wherein PSCs, LIBs, and strain sensors were combined in a flexible assembly. A wearable PSC-LIB-driven photo-rechargeable lithium-ion strain sensor showed a PCE of 8.41% and an output voltage of 3 V at a discharge current density of 0.1 A g^−1^. The device could achieve a significantly high PCE of approximately 6% at a high current density of 1 A g^−1^, which is one of the highest reported performances for photo-charging power sources.

## 3. Conclusions

We have reviewed the development of Pb-free Sn-PSCs. By studying the progress of the Sn-PSC technology, we noticed that the enhancement of *V*_OC_ played an important role in obtaining a high PCE. This was generally achieved by tuning the energy levels of Sn-based perovskites and charge-transporting layers. The Fermi level and bandgap of Sn-based perovskites were controlled by using different chemical compositions. Different electron-transporting layers, such as fullerene derivatives, were explored to find a material that matched the energy levels of the Sn-based perovskite materials. The absorption range could be widened by tuning the bandgap of the Sn-based perovskite layer; however, this could result in reductions in *V*_OC_ and FF. In addition, the increase in *J*_SC_ did not match the decrease in *V*_OC_ and FF. Furthermore, passivation and protection of Sn^2+^ from oxidation are important for increasing the FF and device stability. Various chemical compounds were used as additives. Further, higher PCEs for Sn-PSCs can potentially be achieved by investigating unexplored hole-transporting materials. High-performance Sn-PSCs are limited to inverted architectures and PEDOT:PSS hole-transporting layers. However, the highest PCEs have been reported for the normal architecture of Pb-based PSCs; thus, investigating methods for improving the performance of the normal architecture of Sn-PSCs is crucial. In this context, it is important to inhibit the damage caused by spiro-MeOTAD and its components. To this end, carbon nanotube electrodes which are laminated before the application of spiro-MeOTAD can be used [11,57,58,59,60]. Another method is to apply poly(triarylamine) (PTAA), which has yielded relatively high PCEs. The challenge in utilising PTAA lies in its wettability. A Sn-based perovskite precursor that contains DMSO cannot sufficiently cover the surface of PTAA as the latter is non-protic polar and highly dielectric. Modifying the PTAA surface could be a good solution for enhancing its applicability in Sn-PSCs. Overall, we reviewed Pb-free Sn-based perovskite solar cells in a chronological order as there has not been a paper describing the progress of the technology from the historical point of view. From the nascence of Sn-PSC, the strategy transitioned from single cation perovskites to the mixed cation approach, which involved doping by additives and formation of two-dimensional perovskite layer. Later, the deposition method became critical. The shift in the focus of science in Sn-PSCs is clearly categorised in each title of the subsection. The reported PCE of Sn-PSCs is now sufficiently high for their use in photo-charging systems. The low *V*_OC_ and *J*_SC_ values can be overcome by connecting many devices in series or in parallel. Furthermore, a printed circuit board interface, with an electrical algorithm, can be employed between the Sn-PSCs and LIBs to better match the voltage and current. From the perspective of commercialisation, stability is much more important than the device PCE. Most of the Sn-PSCs discussed in this review are organic-inorganic mixed perovskites. However, for an actual application, for example, solar roofs, all-inorganic Sn-PSCs might be more applicable owing to their high device stability at the expense of low PCE. [61] Although many facets of Sn-PSCs need to be improved, we believe that Sn-PSCs have the potential to replace Pb-PSCs, considering their rate of development. We can expect researchers in the field Pb-free PSCs to come up with novel strategies [62] and adoption of new materials towards optimal energy alignments in Sn-PSCs [63] in near future. In the quest for environmentally sustainable society, eco-friendly Sn-PSCs-based energy system this paper discusses will pave the pathway towards greener society, far cry from the energy crisis.

## Figures and Tables

**Table 1:** Record table containing the reported Sn-PSCs and the corresponding photovoltaic parameters mentioned in Section 2.1.

Report	Year [Ref.]	Device Structure	*J*_SC_ [mA m^−2^]	*V*_OC_ [V]	FF	PCE [%]
A	2016 [26]	Glass/ITO/PEDOT:PSS/FASnI_3_/C_60_/BCP/Ag	22.07	0.47	0.60	6.22
B	2016 [27]	Glass/FTO/c-TiO_2_/m-TiO_2_/MASnI_3_/PTAA/Au	19.92	0.38	0.51	3.86
C	2016 [28]	Glass/FTO/c-TiO_2_/m-TiO_2_/FASnI_3_/spiro-MeOTAD/Au	23.70	0.32	0.63	4.80
D	2017 [29]	Glass/FTO/c-TiO_2_/m-TiO_2_/{en}FASnI_3_/spiro-MeOTAD/Au	22.54	0.48	0.66	7.14

Note: ITO, indium tin oxide; FTO, fluorine-doped tin oxide.

**Table 2:** Record table containing the reported Sn-PSCs and the corresponding photovoltaic parameters mentioned in Section 2.2.

Report	Year [Ref.]	Device Structure	*J*_SC_ [mA m^−2^]	*V*_OC_ [V]	FF	PCE [%]
E	2017 [30]	Glass/ITO/PEDOT:PSS/FA_0.75_MA_0.25_SnI_3_/ C_60_/BCP/Ag	21.20	0.61	0.63	8.12

**Table 3:** Record table containing the reported Sn-PSCs and the corresponding photovoltaic parameters mentioned in Section 2.2.1.

Report	Year [Ref.]	Device Structure	*J*_SC_ [mA m^−2^]	*V*_OC_ [V]	FF	PCE [%]
F	2018 [31]	Glass/ITO/PEDOT:PSS/FASnI_3_ (PEAI)/C_60_/ BCP/Ag	24.1	0.53	0.71	9.00

**Table 4:** Record table containing the reported Sn-PSCs and the corresponding photovoltaic parameters mentioned in Section 2.3.

Report	Year [Ref.]	Device Structure	*J*_SC_ [mA m^−2^]	*V*_OC_ [V]	FF	PCE [%]
G	2018 [32]	Glass/ITO/PEDOT:PSS/FASnI_3_/C_60_/BCP/Ag	22.45	0.47	0.68	7.09

**Table 5:** Record table containing the reported Sn-PSCs and the corresponding photovoltaic parameters mentioned in Section 2.4.

Report	Year [Ref.]	Device Structure	*J*_SC_ [mA m^−2^]	*V*_OC_ [V]	FF	PCE [%]
H	2018 [34]	Glass/ITO/PEDOT:PSS/FASnI_3_ (1%EDAI_2_)/C_60_/BCP/Ag	21.30	0.58	0.72	8.9
I	2018 [35]	Glass/ITO/PEDOT:PSS/FA_0.75_MA_0.25_Sn_1-x_Ge_x_I_3_/C_60_/BCP/Ag	21.90	0.45	0.70	6.90
J	2018 [36]	Glass/ITO/PEDOT:PSS/FA_0.75_MA_0.25_SnI_3_/ C_60_/BCP/Ag	19.40	0.55	0.61	7.20
K	2019 [37]	Glass/ITO/PEDOT:PSS/GA_x_FA_0.98-x_SnI_3_ (1%EDAI_2_)/C_60_/BCP/Ag	21.20	0.62	0.73	9.60
L	2019 [38]	Glass/ITO/PEDOT:PSS/FASnI_3_-PVA/C_60_/BCP/Ag	20.37	0.63	0.69	8.92

**Table 6:** Record table containing the reported Sn-PSCs and the corresponding photovoltaic parameters mentioned in Section 2.5.

Report	Year [Ref.]	Device Structure	*J*_SC_ [mA m^−2^]	*V*_OC_ [V]	FF	PCE [%]
M	2019 [39]	Glass/FTO/PCBM/CsSn_0.5_Ge_0.5_I_3_/spiro-MeOTAD/Au	18.61	0.63	0.61	7.11

**Table 7:** Record table containing the reported Sn-PSCs and the corresponding photovoltaic parameters mentioned in Section 2.6.

Report	Year [Ref.]	Device Structure	*J*_SC_ [mA m^−2^]	*V*_OC_ [V]	FF	PCE [%]
N	2019 [40]	Glass/FTO/PEDOT:PSS/FA_0.98_EDA_0.01_SnI_3_/C_60_/BCP/Ag/Au	23.09	0.60	0.73	10.18
O	2020 [41]	Glass/ITO/PEDOT:PSS/FASnI_3_-FBH/C_60_/BCP/Ag	21.10	0.60	0.75	9.47
P	2020 [42]	Glass/FTO/Cu-NiO_x_/FASnI_3_-4AMP /PCBM/BCP/Ag	21.15	0.69	0.74	10.86
Q	2020 [43]	Glass/FTO/PEDOT:PSS/CsFASnI_3_/PCBM/BCP/Ag	21.60	0.64	0.75	10.40
R	2020 [44]	Glass/ITO/PEDOT:PSS/FA_0.75_MA_0.25_SnI_3_/PCBM/C_60_/BCP/Ag	22.0	0.76	0.69	11.50

**Table 8:** Record table containing the reported Sn-PSCs and the corresponding photovoltaic parameters mentioned in Section 2.7.

Report	Year [Ref.]	Device Structure	*J*_SC_ [mA m^−2^]	*V*_OC_ [V]	FF	PCE [%]
S	2020 [50]	Glass/ITO/PEDOT:PSS/PEA15-SCN FASnI_3_ /ICBA/BCP/Ag	17.40	0.94	0.75	12.40
T	2020 [51]	Glass/FTO/PEDOT:PSS/Ge doped FA_0.98_EDA_0.01_SnI_3_ (EA 0.1)/ C_60_/BCP/Ag/Au	20.32	0.84	0.78	13.24
U	2021 [52]	Glass/ITO/PEDOT:PSS/FA_0.8_GA_0.2_SnI_3_/BAC_2_SnI_4_/C_60_/BCP/Ag	21.1	0.65	0.76	10.60
V	2021 [53]	Glass/ITO/PEDOT:PSS/FASnI_3_ (PAI)/C_60_/BCP/Ag	22.48	0.77	0.70	12.11
